# Trimethylamine N-oxide promotes demyelination in spontaneous hypertension rats through enhancing pyroptosis of oligodendrocytes

**DOI:** 10.3389/fnagi.2022.963876

**Published:** 2022-08-22

**Authors:** Xiaotan Ji, Long Tian, Shenna Niu, Shumei Yao, Chuanqiang Qu

**Affiliations:** ^1^Department of Neurology, Shandong Provincial Hospital, Shandong University, Jinan, China; ^2^Department of Neurology, Jining No. 1 People’s Hospital, Jining, China; ^3^Department of Neurology, Shandong Provincial Hospital Affiliated to Shandong First Medical University, Jinan, China

**Keywords:** trimethylamine N-oxide, white matter lesions, demyelination, hypertension, pyroptosis

## Abstract

**Background:**

Hypertension is a leading risk factor for cerebral small vessel disease (CSVD), a brain microvessels dysfunction accompanied by white matter lesions (WML). Trimethylamine N-oxide (TMAO), a metabolite of intestinal flora, is correlated with cardiovascular and aging diseases. Here, we explored the effect of TMAO on the demyelination of WML.

**Methods:**

Spontaneous hypertension rats (SHRs) and primary oligodendrocytes were used to explore the effect of TMAO on demyelination *in vivo* and *in vitro*. T2-weighted magnetic resonance imaging (MRI) was applied to characterize the white matter hyperintensities (WMH) in rats. TMAO level was evaluated using LC-MS/MS assay. The histopathological changes of corpus callosum were measured by hematoxylin-eosin and luxol fast blue staining. And the related markers were detected by IHC, IF and western blot assay. Mito Tracker Red probe, DCFH-DA assay, flow cytometry based on JC-1 staining and Annexin V-FITC/PI double staining were conducted to evaluate the mitochondrial function, intracellular ROS levels and cell apoptosis.

**Results:**

SHRs exhibited stronger WMH signals and a higher TMAO level than age-matched normotensive Wistar-kyoto rats (WKY). The corpus callosum region of SHR showed decreased volumes and enhanced demyelination when treated with TMAO. Furthermore, TMAO significantly elevated ROS production and induced NLRP3 inflammasome and impairment of mitochondrial function of oligodendrocytes. More importantly, TMAO enhanced the pyroptosis-related inflammatory death of oligodendrocytes.

**Conclusion:**

TMAO could cross the blood-brain barrier (BBB) and promote oligodendrocytes pyroptosis *via* ROS/NLRP3 inflammasome signaling and mitochondrial dysfunction to promote demyelination, revealing a new diagnostic marker for WML under hypertension.

## Introduction

Cerebral small vessel disease (CSVD) is a common neurological disorder caused by various etiologies factors, contributing to brain microvessels and small arteries dysfunction (Li Q. et al., 2018). CSVD is characterized by a series of neuroimaging manifestations of recent small subcortical infarcts, white matter hyperintensities (WMH), microbleeds, lacunes, enlarged perivascular spaces and cerebral atrophy, which are considered to be the main vascular factors leading to dementia, cognitive impairment, gait disturbance and stroke (Li Q. et al., 2018; Cannistraro et al., 2019). Mounting evidences indicated that CSVD is mainly attributed to aging and vascular risk factors including diabetes, hypertension and amyloidosis. Among these, hypertension is the focus of global public health and a risk factor for CSVD. Patients with long-term hypertension are prone to induce small vascular disease (Petrea et al., 2020).

WMH, also called white matter lesion (WML) and detected by T2-weighted magnetic resonance imaging (MRI), is a cerebral structural change characterized by white matter demyelination. White matter is mainly composed of myelinated nerve fibers and myelin-producing glial cells. Therein, myelinated nerve fibers are surrounded by myelin sheaths produced by oligodendrocytes in the central nervous system (Nave and Werner, 2014). Reports revealed that the development of hypertension could induce oligodendrocyte loss and demyelination (Jalal et al., 2012; Cui et al., 2021). In addition, Myelin loss attributed to oligodendrocyte death was associated with neuroinflammation in hypertensive rats (Jalal et al., 2012). Recent studies demonstrated that pyroptosis was involved in oligodendrocyte injury and inflammatory demyelination (Loda and Balabanov, 2012; Yang et al., 2020; Kiasalari et al., 2021). Hereon, investigating whether the oligodendrocyte loss in hypertension-induced demyelination was related to pyroptosis and the associated molecules as well as the underlying mechanism provided a novel insight into a new therapeutic strategy for hypertension-induced WML.

Gut microbiota exhibits an important role in the regulation of metabolic, nervous and immune systems (Morais et al., 2021). In addition, increasing studies have reported that microbiota is associated with a series of diseases, including Alzheimer’s disease, atherosclerosis, type-II diabetes and hypertension (Forslund et al., 2015; Chen M. W. et al., 2017; Jonsson and Backhed, 2017; Arrona Cardoza et al., 2021). Gut microbiota-brain axis is involved in the pathogenesis of neurological disorders. For example, intestinal microbiota metabolites could regulate the development of the myelination process (Hoban et al., 2016; Ntranos and Casaccia, 2018; McMurran et al., 2019; Keogh et al., 2021). Gut microbiota metabolites mainly consist of lipopolysaccharides (LPS), short-chain fatty acids (SCFA) and trimethylamine-N-oxide (TMAO), affecting cardiovascular and cerebral health (Parker et al., 2020).

TMAO is one of the main metabolites of gut microbiota. Studies have found that TMAO levels are closely associated with cardiovascular disease and neurodegenerative diseases (Wang et al., 2011; Tang et al., 2015; Chen et al., 2020). For instance, TMAO can affect the brain aging process and aggravate aging-related cognitive dysfunction in aged mice (Li D. et al., 2018). Gut microbiota-dependent TMAO is involved in the severity and progression of Parkinson’s disease (Chen et al., 2020). A recent meta-analysis result has shown that high plasma TMAO concentration was positively correlated with the risk factor of hypertension (Ge et al., 2020). And obstructive sleep apnea combined with a high salt diet results in an increase in blood TMAO levels and a reduction of anti-inflammatory factors, contributing to the aggravation of hypertension in rats (Liu et al., 2019), showing that the increased TMAO levels may be associated with the development of hypertension to some extent. Besides, the high TMAO level is positively correlated with the severity of WMH signal in cerebral small vessel imaging (Chen et al., 2021). And accumulating studies have shown that TMAO can cross the blood-brain barrier (BBB) and exert a crucial role in cerebrospinal fluid (CSF) (Vernetti et al., 2017; Vogt et al., 2018). Moreover, TMAO treatment results in oxidation stress, mitochondrial impairments and neuroinflammation in hippocampus region aging model animals (Li D. et al., 2018, Zhao et al., 2019). Based on these facts, we wondered about the effect and mechanism of TMAO on demyelination in hypertension-induced WML.

In the present study, we explored whether TMAO treatment could induce pyroptosis of oligodendrocytes, contributing to white matter demyelination in hypertension. For this purpose, we treated spontaneous hypertension rats (SHR), a hypertensive CSVD rat model *in vivo*, and oligodendrocytes *in vitro* with TMAO, respectively. We found that SHR administrated with TMAO orally developed the aggravated demyelination in white matter *in vivo*, and TMAO treatment-induced oligodendrocyte pyroptosis *in vitro*.

## Materials and methods

### Animals

All animal experiments were approved by the Ethics Committee for laboratory animal of Shandong Provincial Hospital and carried out on the basis of the guide published by National Institutes of Health. Adult male 24- and 35-week SHR and age-matched normotensive Wistar-Kyoto rats (WKY) were used in this research. All rats were purchased from Charles River Laboratory Animal Technology Co., Ltd. (Beijing, China), which were free access to water and food under a feeding environment with 12-h light-dark cycle.

For analysis of the change of WML and TMAO levels in hypertension development, 24- and 35-week SHR and age-match WKY rats (*n* = 6/group) were utilized for T2-weighted MRI imaging and TMAO detection. Additionally, for analysis of TMAO function, 24-week SHR and age-matched WKY rats were divided into 4 groups at random, respectively (*n* = 6/group): WKY + water, WKY + TMAO, SHR + water and SHR + TMAO. Rats in TMAO group were fed drinking water with 333 mg/L of TMAO applied (Sigma-Aldrich, St. Louis, MO, United States) for 11 weeks. And rats in water group drank sterile water for 11 weeks. After T2-weighted MRI, rats were euthanized to collect the plasma, CSF and brain tissues for subsequent experiments.

### Magnetic resonance imaging

MRI imaging was conducted on a 9.4-Tesla Bruker Biospec 94/20 USR scanner with a surface coil for brain imaging and a dedicated rat head coil. Rats were anesthetized with 2.5% isoflurane using an induction chamber and then placed on MRI cradle in prone position. A bite bar was used to immobilize the head to prevent the movement of head and the rats were maintained under anesthesia with inhalation of isoflurane during the scanning process.

The turbo-spin echo sequence parameters for T2-weighted MRI in coronal direction were as follows: TR/TE = 4,000/45 ms, matrix size = 256 × 256, echotrain length ETL = 7, field of view (FOV) = 35 × 35 mm^2^, slice thickness = 1 mm. The segmented region of brain structures was manually acquired and analyzed on the multi-slice coronal T2 images with ITK-SNAP software (Yushkevich et al., 2006).

### Trimethylamine N-oxide measurement

Liquid chromatography with triple-quadrupole mass spectrometry (LC-MS/MS) was carried out to determine the concentration of TMAO in plasma and CSF of rats as previous described (Ufnal et al., 2014).

### H&E staining

Paraformaldehyde-fixed and paraffin-embedded specimens were cut into 5 μm thickness, followed by deparaffinization and graded rehydration. The sections were stained with hematoxylin and eosin. The histologic images of white matter were captured using a light microscope (Olympus, Tokyo, Japan).

### Luxol fast blue staining

The deparaffinized sections were hydrated to 95% alcohol, followed by incubation in 0.1% LFB solution (Sigma-Aldrich) at 60°C overnight. After rinsing off excess stain with 95% alcohol and rinsing in distilled water, the sections were differentiated for 30 s in the lithium carbonate solution and counterstained for 40 s in cresyl violet solution. After rinse and differentiation, the sections were mounted with resinous medium. The demyelinated regions appeared in white color and the myelinated regions emerged in blue. The histological changes of corpus callosum region were observed and analyzed under light microscopy.

### Immunohistochemical and immunofluorescence staining

For Immunohistochemical staining, the 5 μm-thickness sections were deparaffinized and rehydrated using a series of grade alcohol. The sections were immersed and stewed in Citrate Antigen Retrieval Solution (Beyotime, Beijing, China) to expose antigen and in 3% hydrogen peroxide solution to eliminate the non-specific staining. After blocking using 5% goat serum, the sections were incubated with primary antibody against MBP (1:1,200 diluted, Abcam, United Kingdom) at 4°C overnight. Next, the HRP-labeled secondary antibody was used for incubation, and then the sections were incubated using DAB substrates for chromogenic reaction. Hematoxylin was employed to stain the nuclear. The neutral resin-mounted sections were visualized under light microscopy.

For immunofluorescence, the 5 μm-thickness sections were permeabilzed with Triton X-100 for 1 h and stained using mouse anti-MBP antibody (1:50; Cell Signaling Technology, United States) and rabbit anti-GSDMD antibody (1:50; CST) at 4°C overnight. Then, the sections were incubated with the goat anti-mouse IgG/PE-labeled and goat anti-rabbit IgG/FITC-labeled secondary antibodies at darkness. For oligodendrocytes treated with TMAO, cells were fixed using 4% paraformaldehyde and permeabilzed with Triton X-100, and incubated with TUNEL (Beyotime) and caspase-1 rabbit polyclonal antibody (1:50; Proteintech, United States) at 4°C overnight and Alexa Fluor 488-conjugated Goat Anti-Rabbit IgG(H + L). DAPI (blue) was used to stain neuclus Anti-fluorescence attenuation mounting medium (Solarbio, Beijing, China) was used to mount the sections and the fluorescence result was observed under the fluorescence microscopy (Leica, Germany).

### Cell culture

Primary oligodendrocytes were purchased from Procell Life Science and Technology Co., Ltd. (Wuhan, China) and cultured with oligodendrocytes complete medium (Procell, Wuhan, China) in an incubator with a humidity of 95%, a CO_2_ concentration at 5% and the temperature of 37°C. After being treated with TMAO for 48 h, oligodendrocytes were collected for the following analysis.

### Western blot assay

Total proteins were extracted with RIPA lysis buffer. A protein quantification kit (Sigma-Aldrich) was used for quantification of the protein concentration. Isolated proteins using 10% SDS-PAGE were transferred into PVDF membranes (Millipore, United States). And then the membranes were blocked using 5% non-fat milk, followed by incubation with primary antibodies against MBP (1:1,200, CST, United States), NLRP3 (1:1,000, CST), IL-1β (1:1,200, R&D system, United States), IL-18 (1:1,000, R&D system), cleaved-caspase 1 (1:1,000, CST), GSDMD-N (1:1,200, CST), β-actin (1:3,000, CST) overnight at 4°C. Then the membranes were incubated with the HRP-labeled specific secondary antibody for 60 min. The protein bands were observed using ECL reagent. The intensity of bands was analyzed *via* Image J software.

### Enzyme-linked immunosorbent assay

The secretion levels of inflammatory factors (IL-1β and IL-18) in cell supernatant and CSF were examined using commercial enzyme-linked immunosorbent assay (ELISA) kits in accordance with the protocols provided (R&D systems). The ELISA kits detecting the levels of superoxide dismutase (SOD) and malonaldehyde (MDA) were obtained from Elabscience Biotechnology Co., Ltd. (Wuhan, China). A microplate reader was used to measure the optical density (OD) values at 450 nm. The relative protein concentrations were calculated in accordance with the standard curves.

### Determination of reactive oxygen species

The intracellular reactive oxygen species (ROS) production was evaluated using flow cytometry based on 2′, 7′-dichlorodihydrofluorescein diacetate (DCFH-DA) staining. Cells were incubated with 10 μM DCFH-DA at a 37°C incubator for 0.5 h, and the addition of DMSO was as the control group. After washing with HBSS buffer, the DCF positive cells were analyzed and quantified *via* flow cytometry.

### Cell viability

The cell counting kit (CCK-8; Beyotime) was employed to measure the cell viability of oligodendrocytes with the different treatments. cells at a density of 4 × 10^3^/well were seeded in 96-well plates with different concentrations of TMAO (10, 50, 100, 200, 400, 800 μM) for 48 h. 10 μL CCK-8 reagents were added into wells and incubated for 2 h. The OD value was evaluated at a reference wavelength of 630 nm and a test wavelength of 450 nm with a microplate reader.

### Cell apoptosis detection

Annexin V-FITC/PI double staining (Absin, Shanghai, China) was performed to determine the apoptotic cell proportion. 4 × 10^5^ cells per well were seeded into 6-well plates with the different treatments and incubated for 48 h. Then, cells and cell supernatant were harvested and detected by flow cytometry according to the protocol provided.

### Detection of mitochondrial membrane potential

Mitochondrial membrane potential detection Kit (MedChemExpress, Shanghai, China) was employed to examine the mitochondrial membrane potential. Briefly, cells at a density of 4 × 10^5^/well were seeded into 6-well plates with different treatments for 48 h and then harvested to centrifuge at 1,000 rpm for 5 min. Then, 10 μg/mL JC-1 probes were used to incubate the cells at 37°C for 20 min. After washing with non-serum fresh medium three times, cells were detected using flow cytometry and analyzed with FlowJo software.

### Mitochondrial damage detection

Cellular mitochondrial was measured and analyzed using a specific MitoTracker Red staining (ThermoFisher Scientific, Waltham, MA, United States). Cells with a density of 3 × 10^4^/well were seeded into 12-well plates and stained with mito tracker red probe at 37°C for 20 min, and observed under fluorescence microscopy.

### Statistical analysis

All results were expressed as mean ± standard deviation and analyzed statistically using Graphpad prism version 8.0 (Graphpad Software, La Jolla, CA, United States). The association relationship was analyzed by Pearson Correlation Analysis. The difference between two groups was analyzed using Student’s *t*-test. One-way ANOVA followed by Bonferroni’s *post-hoc* test was applied to analyze differences among multiple groups. *P* < 0.05 was deemed as statistical significance.

## Results

### Increased trimethylamine N-oxide promoted white matter lesions in spontaneous hypertension rats

**FIGURE 1:**
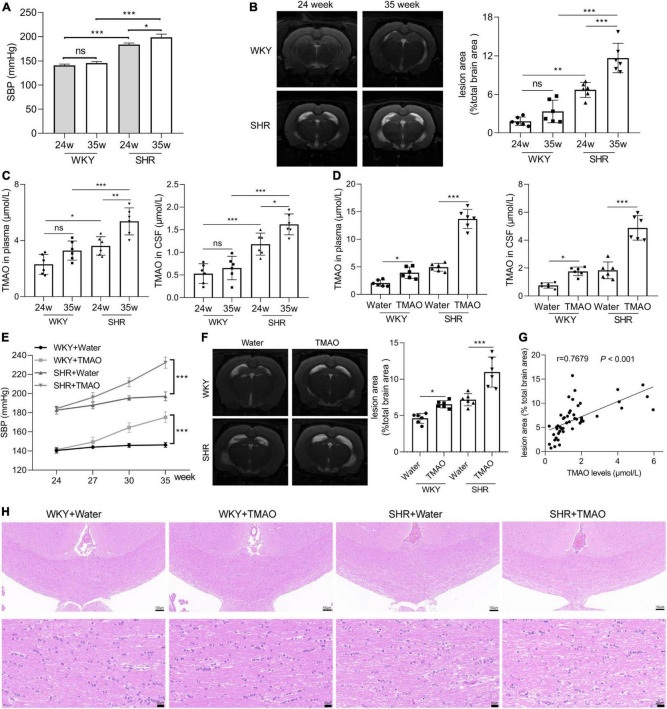
Increased TMAO promoted white matter lesions. **(A)** Systolic blood pressure (SBP) at 24 and 35 week of WKY and SHR (*n* = 6/group). **(B)** Images and quantation of T2-weighted MRI in 24- and 35-week SHR and age-matched normotension WKY rats. **(C)** TMAO level in plasma and CSF detected by LC-MS/MS (*n* = 6/group). 24-week-SHR and age-matched normotensive WKY rats were treated with TMAO for 11 weeks. **(D)** TMAO level in plasma and CSF detected by LC-MS/MS (*n* = 6/group). **(E)** SBP at different ages of WKY and SHR treated with TMAO (*n* = 6/group). **(F)** Images and quantation of T2-weighted MRI in SHR and WKY rats. **(G)** The relationship between TMAO levels and white matter lesion in brain was analyzed using Spearman correlation analysis. **(H)** Changes in white matter injury detected by H&E staining. Scale bar: upper, 100 μm; lower, 20 μm. The data were expressed as mean ± *SD* of three independent experiments. ns, no significance. *P < 0.05, **P < 0.01, ***P < 0.001.

Systolic blood pressure (SBP) was detected in 24- and 35-week old SHR and age-matched normotensive homogeneous WKY rats and the results were shown in Figure 1A. SBP levels in 24- and 35-week old SHR were extremely higher than in age-matched WKY rats, in addition, SBP levels were a little higher in 35-week old SHR than in 24-week old SHR (Figure 1A). Next, in order to explore whether the development of hypertension resulted in the lesion in white matter regions, MRI analysis was carried out. We found that the WMH signal of SHR rats was significantly severe compared with age-matched WKY rats, and 35-week SHR rats developed stronger WMH signals than 24-week SHR rats, indicating that the development of hypertension promoted the severity of WML with age (Figure 1B). TMAO, a gut microbiota metabolite, is reported to be correlated with WMH in CSVD and the development of hypertension (Liu et al., 2019; Chen et al., 2021). Hereon, we detected the change of TMAO concentration in SHR and WKY rats. The result of LC-MS/MS demonstrated that TMAO levels were higher in plasma and CSF of SHR rats than WKY rats, and there was a slight increase in 35-week SHR rats compared with 24-week SHR rats, suggesting that TMAO levels might be associated with the development of hypertension (Figure 1C). To investigate whether TMAO played a role in WML, we treated the 24-week SHR and age-matched normotensive WKY rats with 333 mg/L oral administration of TMAO for 11 weeks. The TMAO treatment significantly increased the TMAO concentration in plasma and CSF, respectively (Figure 1D). And increased TMAO levels significantly enhanced SBP levels in SHR and WKY rats (Figure 1E). The T2-w imaging of MRI showed a severity of WMH in WKY + TMAO and SHR + TMAO groups compared with WKY + Water and SHR + Water groups, respectively (Figure 1F). More intriguingly, Spearman correlation analysis showed that TMAO levels were positively associated with WML area in cerebrum of rats (Figure 1G; *r* = 0.7679, *P* < 0.001). Furthermore, the results of H&E showed that the nerve fibers of white matter in SHR-water group were significantly sparse, disordered arrangement and increased vacuolation compared with WKY + Water group, and TMAO treatment significantly aggravated these pathological features in WKY + TMAO and SHR + TMAO groups (Figure 1H). These data revealed that TMAO could promote WML in SHR.

### Trimethylamine N-oxide resulted in demyelination of white matter in hypertensive rats

**FIGURE 2:**
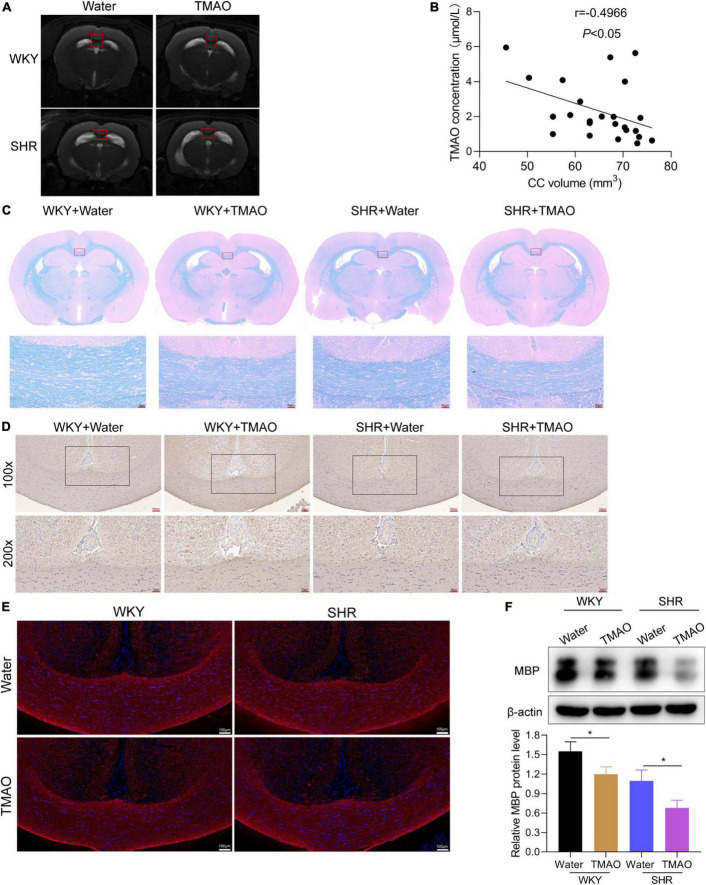
TMAO promoted demyelination of white matter in hypertensive rats. **(A)** Images of T2-weighted MRI in SHR and WKY rats treated with TMAO or water. **(B)** The association between TMAO levels and corpus callosum volumes was analyzed using Spearman correlation analysis. **(C)** Luxol Fast Blue staining was used to evaluate myelin change. Scale bar: 50 μm. **(D)** MBP expressions were measured by IHC staining. Scale bar: upper, 100 μm; lower, 50 μm. **(E)** Immunofluorescence assay for MBP (red) staining in corpus callosum tissues. Scale bar: 100 μm **(F)** MBP expressions were measured by western blot. The data were expressed as mean ± SD of three independent experiments. *P < 0.05.

As previously described, demyelination of cerebral white and gray matter could give rise to neuronal degeneration in a series of neurological disorders, including multiple sclerosis and hypertension-induced CSVD (Kaiser et al., 2014; Hoftberger and Lassmann, 2017). Here, we focused on exploring whether TMAO was involved in the process of demyelination in SHR. As shown in Figure 2A, SHR and WKY rats treated with TMAO exhibited decreased volumes of corpus callosum, a region enriched with myelinated nerve fibers, in contrast to SHR and WKY rats fed with water. The TMAO concentration in CSF showed a negative correlation with the volumes of corpus callosum (Figure 2B; *r* = –0.4966, *P* < 0.05). Besides, the results of LFB staining demonstrated the smaller volumes of corpus callosum and aggravated degree of demyelination accompanied by a dramatic loss of myelin in TMAO-treated SHR (Figure 2C). Afterward, we detected the expression of MBP by immunohistochemistry and western blot. We found a significant decrease of MBP expressions in corpus callosum region of SHR with TMAO treatment, and the same result was discovered in WKY rats as revealed by immunohistochemical, immunofluorescence staining and western blot assay (Figures 2D–F). These data indicated that enhanced TMAO promoted the demyelination of corpus callosum in hypertension rats.

### Trimethylamine N-oxide induced pyroptosis of oligodendrocytes to promote white matter demyelination in spontaneous hypertension rats

**FIGURE 3:**
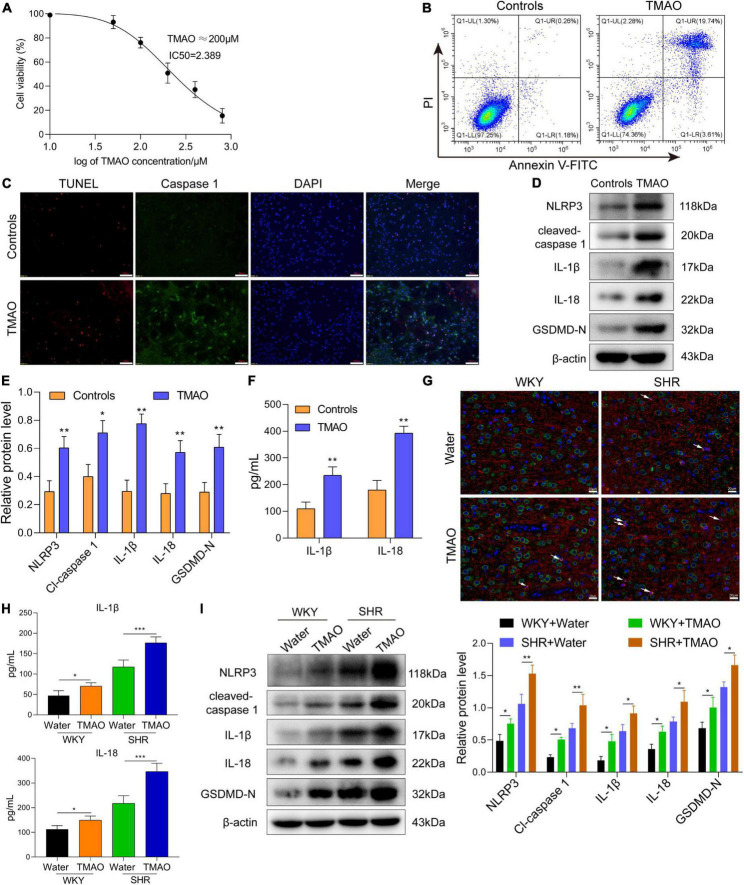
TMAO induced pyroptosis of oligodendrocytes to enhance demyelination in SHR. **(A)** CCK-8 kit was used to measure the cell viability of oligodendrocytes treated with TMAO. **(B)** Flow cytometry based on Annexin V-FITC/PI staining was used to detect the proportion of apoptotic cells. **(C)** Immunofluorescence assay for TUNEL (red) and caspase-1 (green) was carried out to measure the change of cell death. DAPI (blue) was used to stain neuclus. Scale bar = 50 μm. **(D)** The expressions and **(E)** quantitation of NLRP3, IL-1β, IL-18, cleaved-caspase 1 and GADMD-N terminal in cells were detected *via* western blot. **(F)** The levels of IL-1β and IL-18 in conditional medium were detected by ELISA assay. **(G)** GSDMD (green) and MBP (red) double staining immunofluorescence analysis in white matter region was performed. Arrows were for GSDMD^+^MBP^+^ oligodendrocytes in cerebrum. Scale bar = 20 μm. **(H)** The levels of IL-1β and IL-18 in CSF were detected by ELISA assay. **(I)** The expressions of NLRP3, IL-1β, IL-18, cleaved-caspase 1 and GADMD-N terminal were detected *via* western blot. The data were expressed as mean ± *SD* of three independent experiments. *P < 0.05, **P < 0.01, ***P < 0.001.

Oligodendrocyte, the myelin-forming cells in central nervous system, was closely involved in demyelination and remyelination. Hereon, we induced oligodendrocytes with TMAO *in vitro* to observe the function and mechanism of TMAO in oligodendrocytes. The CCK-8 assay displayed a decrease of cell viability with the increase of TMAO concentration, and 200 μM TMAO resulted in 50% decrease of oligodendrocyte survival (Figure 3A). Based on the result of cell viability, TMAO at 200 μM was used for further experiments.

TMAO treatment induced a remarkable enhanced number of late-stage (Annexin V^+^/PI^+^) apoptotic cells (Figure 3B). In addition, we found that TMAO promoted the pyroptotic cell death in oligodendrocytes indicated by immunofluorescence assay based on TUNEL (red) and caspase-1 (green) co-staining (Figure 3C). And the results of western blot assay and ELISA assay showed that the levels of pyroptosis-related proteins and the secretion levels of inflammatory factors (IL-1β, IL-18) were significantly elevated in TMAO group compared with PBS group (Figures 3D–F).

Next, we measured the pyroptosis of oligodendrocytes in cerebrum *via* GSDMD and MBP double-staining immunofluorescence analysis. The result of Figure 3G exhibited that TMAO induced pyroptotic cell death in MBP positive oligodendrocytes, namely GSDMD (green)^+^ MBP (red)^+^ oligodendrocytes. Furthermore, we intended to find whether TMAO affected the demyelination of white matter *in vivo*. Here, we explored whether TMAO induced inflammatory demyelination *via* pyroptosis. As indicated in Figure 3H, the levels of IL-1β and IL-18 in CSF of SHR and WKY rats fed with TMAO showed a significant increase compared with those in CSF of SHR and WKY rats fed with water. In addition, The western blot data in Figure 3I indicated that TMAO promoted the expressions of pyroptosis-related proteins (NLRP3, IL-1β, IL-18, cleaved-caspase 1 and GADMD-N terminal) in white matter region of SHR and WKY rats. These results demonstrated that TMAO could induce the pyroptosis of oligodendrocytes to promote inflammatory demyelination in white matter of SHR.

### Trimethylamine N-oxide promoted oxidative stress through reactive oxygen species production to activate NLRP3 inflammasome to induce pyroptosis of oligodendrocytes

**FIGURE 4:**
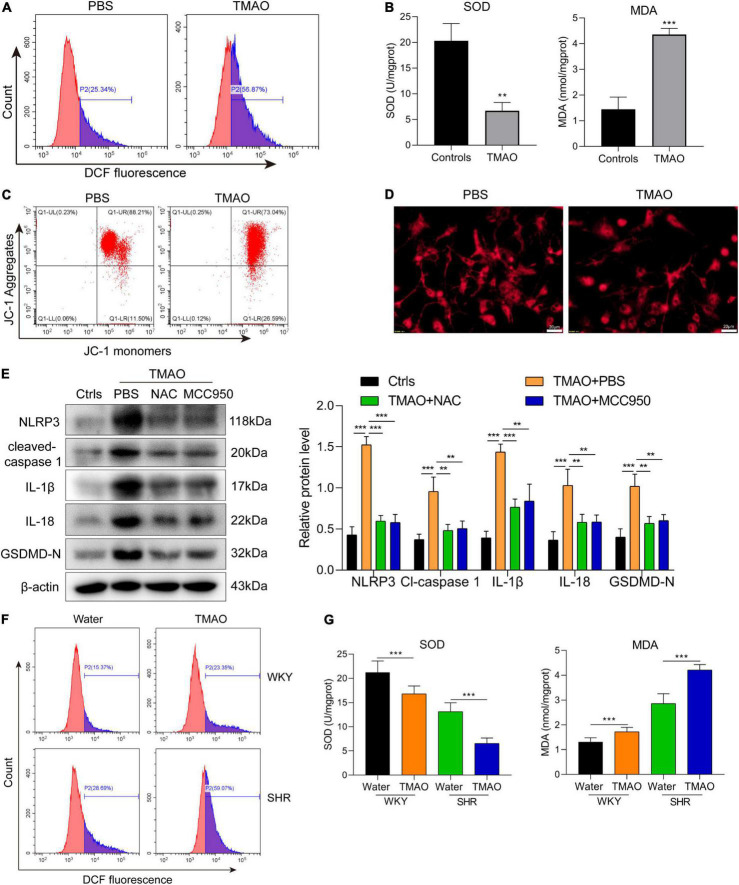
TMAO activated ROS/NLRP3 pathway to induce pyroptosis of oligodendrocytes. **(A)** The ROS level in oligodendrocyte was evaluated *via* DCFH-DA fluorescent probe. **(B)** The levels of SOD and MDA in oligodendrocyte were detected by ELISA assay. **(C)** Flow cytometry based on JC-1 staining was used to detect the change of mitochondrial membrane potential. **(D)** Mito-tracker red probe was used to evaluate the mitochondrial damage of oligodendrocytes *in vitro*. Scale bar = 20μm. **(E)** The expressions of NLRP3, IL-1β, IL-18, cleaved-caspase 1 and GADMD-N terminal in cells were detected *via* western blot. **(F)** The ROS level in cerebrum was evaluated *via* DCFH-DA fluorescent probe. **(G)** The levels of SOD and MDA were detected by ELISA assay. The data were expressed as mean ± SD of three independent experiments. **P < 0.01, ***P < 0.001.

To determine the underlying mechanism of TMAO in promoting oligodendrocyte pyroptosis, we firstly evaluated whether TMAO induced the change of ROS level in oligodendrocytes. As exhibited in Figure 4A, the cell proportion of DCF^+^ oligodendrocytes was remarkably augmented with TMAO treatment. And TMAO suppressed the levels of SOD but enhanced the MDA levels (Figure 4B). The above results demonstrated that TMAO induced intracellular ROS production in oligodendrocytes. Increasing evidences indicated that mitochondrial ROS overproduction could induce oxidative stress, contributing to the development of neurodegenerative disorders (Wu et al., 2019). Here, flow cytometry based on JC-1 staining showed that TMAO inhibited the ratio of Q2/Q3 district, demonstrating that TMAO was involved in the damage of mitochondrial membrane potential (MMP, △Ψ) (Figure 4C). In addition, TMAO-treated oligodendrocytes had increased small mitochondrial fragments and decreased mitochondrial mass compared with PBS-treated oligodendrocytes, indicating that TMAO induced dysfunction of mitochondria in oligodendrocytes (Figure 4D). Next, in order to verify the function of ROS and NLRP3 inflammasome in TMAO-treated oligodendrocytes, we induced these cells with ROS inhibitor NAC and NLRP3 inhibitor MCC950, respectively. And we found that TMAO-increased pyroptosis-related proteins were suppressed by NAC or MCC950 treatment (Figure 4E).

Next, we verified whether TMAO induced oxidation stress in SHR *via* evaluating the ROS level by DCFH-DA fluorescent probe. And we found that ROS levels in cerebrum were significantly higher in SHR + TMAO group and WKY + TMAO group than SHR + water group and WKY + water group (Figure 4F). Moreover, TMAO inhibited the expression of oxidation stress marker SOD but promoted MDA level in SHR, exhibiting a correlation between TMAO and oxidation stress in cerebrum of SHR (Figure 4G). Above all, these data exhibited that TMAO promoted oligodendrocytes pyroptosis *via* mitochondrial dysfunction and ROS/NLRP3 pathway.

## Discussion

Hypertension is the most important cause of white matter degeneration and a risk factor for CSVD. In our study, we found that SHR, a typical animal model for hypertensive CSVD, spontaneously developed the loss of white matter volumes, and cerebrovascular inflammation with increasing age, consistent with the previous reports (Gao et al., 2019; Nelson et al., 2021). White matter is composed of neural networks of nerve fibers, contributing to communication and information exchange between different cognitive cortical areas and motor regions. And nerve fibers are wrapped by myelin formed by oligodendrocytes to act as electrical insulation and prevent the nerve fibers from damage (Sharma et al., 2022). Demyelination in the central nervous system could aggravate the dysfunction of white matter. Here, we found that myelin loss in corpus callosum, the white matter structure in the brain to act as a commissural bridge of axon bundles important for brain function, was exacerbated in SHR with age increasing. And the results of the aggravated demyelination in SHR when compared with WKY rats were similar to the data of the previous study (Kaiser et al., 2014).

Increasing evidences have shown that gut microbiota-brain axis exerts a crucial function in the regulation and development of neurological disorders, immune system and metabolism *via* dynamic bidirectional communication (Dabke et al., 2019; Morais et al., 2021). For instance, the gut microbiota is involved in the regulation of motor deficits and coordination dysfunction in rotenone-induced Parkinson’s disease mice (Bhattarai et al., 2021). In addition, gut-derived microbiota metabolites are implicated in the pathogenesis of demyelination in brain diseases. For example, butyrate, a gut flora metabolite, could inhibit cuprizone-induced demyelination through regulating the differentiation of oligodendrocytes (Chen et al., 2019).

TMAO, a seafood-derived molecule, is reported to impact cardiovascular and cerebrovascular health and diseases, including hypertension (Zhang et al., 2021). Our present study exhibited that the TMAO level was significantly increased in blood and CSF of SHR compared with age-matched normotension WKY rats, similar to the previous result reported (Huc et al., 2018). And we also found a slight increase of TMAO level in 35-week SHR in comparison with 24-week SHR and TMAO was positively involved in the decreased volumes of white matter, indicating that TMAO might be correlated with the development of WML. The increased TMAO level is strongly associated with the severity of WMH (Chen et al., 2021). Govindarajulu et al. (2020) reported that TMAO could enhance endoplasmic reticulum stress to induce impairment in synaptic plasticity, contributing to cognitive deficits in Alzheimer’s model and insulin resistance mouse. In our results, TMAO treatment promoted the decreased of white matter volumes, nerve fiber loosening and demyelination in SHR and WKY rats demonstrated by T2w-MRI imaging, HE and LFB staining. In addition, we found that MBP expression was inhibited with TMAO treatment. Our present study firstly exhibited that TMAO promoted the process of demyelination in SHR.

Inflammatory demyelination in the central nervous system could lead to lesions in cerebral white matter. And the previous study has shown that pyroptosis-dependent oligodendrocytes injury-induced inflammatory demyelination (Loda and Balabanov, 2012). Neuroinflammation and demyelination are modulated by NLRP3 and pyroptosis in experimental autoimmune encephalomyelitis model mice (Kiasalari et al., 2021). A recent report has elucidated that TMAO participates in the regulation of neuroinflammation and cognitive dysfunction with aging (Brunt et al., 2021). Furthermore, TMAO is implicated in vascular endothelial cell pyroptosis in atherosclerosis and periodontitis (Wu et al., 2020; Zhou et al., 2021). Hereon, on basis of these previous studies, we speculated a functional role of TMAO-induced pyroptosis in oligodendrocytes for demyelination of white matter, verified by treating SHR *in vivo* and oligodendrocytes *in vitro* with TMAO. And we found that TMAO promoted oligodendrocyte pyroptosis and inflammation in hypertensive rats through the results of TMAO-increased pyroptosis-related protein expressions and the elevated inflammatory factors *in vivo* and *in vitro* for the first time.

Oxidative stress, induced by an imbalance between the production and accumulation of ROS in cells and tissues and the antioxidant defense systems, is a regulatory element in aging and neurological disorders. Excess ROS production can result in the dysfunction of mitochondria. And generated ROS and mitochondrial damage is capable to result in the cell death and dysfunction of oligodendrocytes in corpus callosum region (Clarner et al., 2012; Yeung et al., 2021). Increasing evidences indicated that TMAO is associated with oxidative stress, mitochondria-dependent ROS production and inflammation (Chen M. L. et al., 2017; Meng et al., 2019). Here, we detected whether TMAO could affect ROS generation and mitochondria function *via* DAFH-DA probe, JC-1 staining and mito-tracker red probe in oligodendrocytes, and we exhibited that TMAO enhanced intracellular ROS production and dysfunction of mitochondria to exacerbate the pyroptosis in oligodendrocytes, indicating that TMAO could enhance oxidative stress through modulating mitochondria dysfunction and cellular ROS production. And TMAO-increased pyroptosis-related protein expressions were reversed with NLRP3 inhibitor and ROS inhibitor, revealing that TMAO enhanced inflammatory demyelination *via* NLRP3/ROS modulated pyroptosis in oligodendrocytes. These results in our present study demonstrated the function and mechanism of TMAO in demyelination of white matter regions in SHR. Besides, it is well-known that disruption of BBB integrity is critical to WML in brain. A recent study has uncovered that gut microbiota is involved in the disruption of BBB integrity in spontaneously hypertension stroke prone rats (Nelson et al., 2021). However, the role of TMAO in the BBB integrity of SHR remains to investigate. Therefore, we intended to explore the function of TMAO in BBB integrity of hypertension-induced WMLs in the future study.

## Conclusion

In conclusion, our study revealed the promotive role of TMAO in demyelination of corpus callosum regions, contributing to lesions in white matter of spontaneously hypertensive rats. And TMAO mainly resulted in the pyroptosis of oligodendrocytes to promote the development of demyelination through mitochondrial dysfunction and ROS/NLRP3 pathway. These findings might identify the potential targets for therapeutic intervention and provide new insight into the effect of TMAO in hypertension-induced WML.

## Data availability statement

The datasets used in this present study are available from the corresponding author upon reasonable request.

## Ethics statement

The animal study was reviewed and approved by the Animal Care and Ethics Committee of Shandong Provincial Hospital.

## Author contributions

CQ conceived and designed the project. XJ and LT investigated and performed the experiments and analyzed the data. SN and SY drafted the manuscript. All authors modified and revised the manuscript, final approval of the version to be published, and agreed to be accountable for all aspects of the work.

## Abbreviations

TMAO: Trimethylamine N-oxide
WML: white matter lesion
SHR: spontaneous hypertension rats
WMH: white matter hyperintensities
LFB: luxol fast blue
MBP: myelin basic protein
CSVD: cerebral small vessel disease
CC: corpus callosum
CSF: cerebrospinal fluid
SCFA: short-chain fatty acids
TR: repetition time
TE: echo time
ETL: echo-train length
SOD: superoxide dismutase
MDA: malonaldehyde
LC-MS/MS: Liquid chromatography with triple-quadrupole mass spectrometry
DMSO: dimethyl sulfoxide
ANOVA: analysis of variance.
